# Nesting of *Ceratina nigrolabiata*, a biparental bee

**DOI:** 10.1038/s41598-021-83940-4

**Published:** 2021-03-03

**Authors:** Michael Mikát, Eva Matoušková, Jakub Straka

**Affiliations:** Department of Zoology, Faculty of Science, Charles University, Prague, Czech Republic

**Keywords:** Behavioural ecology, Social evolution

## Abstract

Biparental care is very rare in insects, and it was well-documented in only one bee species to this date – *Ceratina nigrolabiata*. However, biparental care was only recently discovered in this species, and detailed description of natural history of this species is missing. Here, we describe the nesting cycle of *C. nigrolabiata*. Pairs of *C. nigrolabiata* are established before female starts offspring provisioning. After provisioning is finished (when youngest offspring reached larval stage), the male abandons the nest. Males which are present in nests where female already finished provisioning brood cells, are probably mainly temporary visitors. The female can perform long-time offspring guarding, but only 22% of completely provisioned nests are guarded by a female. Most nests (54%) are closed and abandoned, when provisioning is completed, and other (24%) are orphaned before provisioning is finished. Guarded nests have statistically higher number of brood cells provisioned than unguarded nests. Generally, *C. nigrolabiata* is unique among bees due to its biparental behavior, but it has also uncommon traits of nesting biology among *Ceratina* bees, e.g. fast offspring development in comparison with provisioning rate, and high proportion of nests which are closed and abandoned by mother.

## Introduction

Parental care is a very effective way of increasing offspring survival^1–4^. However, it has also significant costs, because care is time-consuming for the parent^1,5^. There is a trade-off between a number of offspring and their survival^6,7^. Parental care results in decreased number of offspring and increased chance of offspring survival^3,8,9^. Moreover, there is strong trade-off between investing in care or in future reproduction^1,10^. Therefore, mating strategies of males and females substantially influence patterns of parental care^11–13^.

Parental care is highly diverse, organisms differ in the length of performed care, in the amount of care invested, and in the behavior connected with parental care^3,14^. Moreover, care can be performed by only male, only female, or both parents^15,16^.

In insects which care about offspring, maternal care is the most common^3,15^. Paternal or biparental care is generally rare in insects. The well-studied examples of biparental care in insects are cockroaches of the genus *Cryptocercus*, and burying, passalid, and bark beetles^12,14^. Insects with biparental care commonly inhabit nutritionally rich resources, for which both interspecific and intraspecific competition is high^3,12^. Therefore, the cooperation of multiple individuals presents an efficient defense of the resource^3^. Most biparental insects build hidden nests in decaying wood or underground^12^. These hidden nests limit the possibility of extra-pair copulations^12^. Usually, females and males differ in their roles, and females usually invest more in offspring^17–19^.

Intensive parental care is typical for aculeate Hymenoptera and usually consist of nest-making and provisioning of offspring with food (pollen or arthropods)^3,20^. Maternal care is the most common type of care in aculeate Hymenoptera, but eusociality evolved several times, and the most prominent aculeate species are eusocial^20–22^. Therefore, aculeate Hymenoptera are an important model taxon for studying the evolution of eusociality, which is derived from maternal care and therefore based on cooperation between females^22,23^. Male participation in parental care is very rare in Aculeata. Males perform care in several eusocial societies, such as bumblebees (e.g. *Bombus griseocollis*) or Polistinae wasps (e.g. *Polistes metricus* and *Ropalidia marginata*), however, their role is usually small^24–28^. Males have a significant role in several species of crabronid wasps of the genus *Trypoxylon,* where biparental care is documented^29,30^. Males of this genus perform nest guarding against natural enemies and also help with nest building^30,31^.

*Ceratina nigrolabiata* has very unusual patterns of care. This species is the only known biparental bee^32^. The female performs brood provisioning, and the male performs nest guarding^32^. Although the female invests more in care, the male’s presence increases nesting productivity^32^. Generally, all small carpenter bees of the genus *Ceratina* nest in broken twigs with soft pith^20,33^. In the beginning of season, a female excavates a burrow^34^. Later, she provisions brood cells; brood cells are linearly arranged, the innermost brood cell contains the oldest offspring and the outermost contains the youngest offspring^33,34^. After finishing provisioning, the mother usually guards her offspring until adulthood^34,35^. When offspring matures, mother feeds them pollen and nectar^36^. However, caring for offspring after provisioning is not obligate for all *Ceratina* bees. *Ceratina* species of the same subgenus as *C. nigrolabiata* (*C. chalybea* and *C. chalcites*) perform facultative nest guarding^35,37^. A female can guard her nest until adulthood and feed her offspring or close the nest by a filling plug and abandon it^35,37^.

In our previous paper^32,^ we reported the presence of biparental care in *C. nigrolabiata* and evaluated benefits of males and females through the phases of provisioning of brood cells. Here we describe the natural history of *C. nigrolabiata* through the whole nesting cycle.

To describe the natural history of parental care in *Ceratina nigrolabiata* we recorded: (1) the phenology of nest types present; (2) the presence of parents at nests of different stages, in particular observing the timing of male care and the paternity of offspring; (3) the consequences of different nesting strategies, including differences in mortality and parasitism between guarded and unguarded nests.

## Results

### Phenology

**Figure 1:**
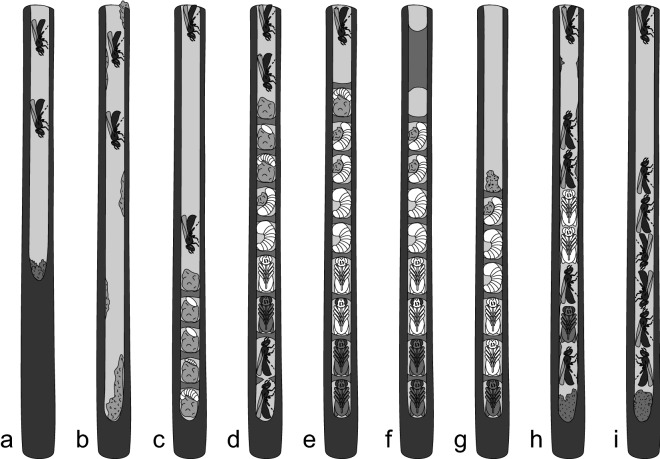
Nesting cycle of *C. nigrolabiata*. (**a**) newly excavated nests—burrow which contains only adult(s) and sometimes fillings. (**b**) discarded nest—burrow where previous nest was discarded, and there are pollen remnants on the walls (**c**) active brood nest—nest in phase brood cell provisioning (**d**) large active brood nest, where egg is present at the top, but young adults already developed at the bottom of nest (**f**) guarded full brood nest—mother guards this nest (**f**) plugged full brood nest—nest is unguarded and closed by a thick filling plug (**g**) orphaned full brood nest—last brood cell partition is thin and above it is commonly pollen from incompletely provisioned brood cell (**h**) full-mature brood nest—this nest contains juveniles, young adults, and sometimes mother (**i**) mature brood nest—this nest contains young adults and sometimes mother. All these figures are hypothetical examples, they are not based on concrete dissected nests.

**Figure 2:**
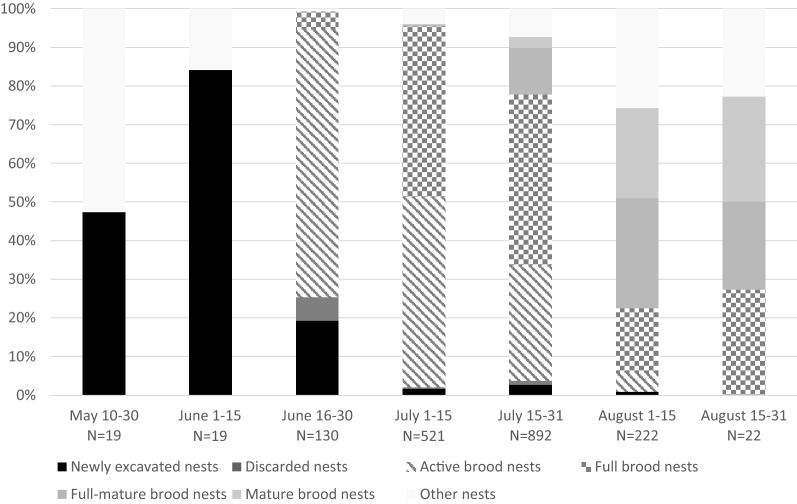
Phenology of *C. nigrolabiata* through nesting season.

*Ceratina nigrolabiata* excavate new nests mainly in May and June, however, some newly excavated nests were also recorded later in the season (Figs. 1, 2). Active brood nests (Table 1) occurred from half of June and appeared in high proportion through whole July. First full brood nests first occurred at the end of June, but the main peak of full brood nests was in July. Full brood nests were also frequent in August. Full-mature and mature brood nests occurred from the end of July, and they were very frequent through August. Other types of nests occurred mainly in the beginning and at the end of season. At the beginning of the season occurred mainly old hibernacula or adults of *C. nigrolabiata* visiting nests of other *Ceratina*. In the late phases of season occurred abandoned nests with only parasites and newly excavated burrows for hibernation.

**Table 1:** Criteria for classification of nest stages.

Nest stage	Offspring present	Stage of the youngest offspring	Brood cell partitions preserved	What female probably does when present
Newly excavated nest	No	NA	NA	Excavating nest or waiting
Discarded nest	No	NA	NA	Discarding previous nest or waiting
Active brood nest	Yes	Egg	Yes	Provisioning brood cells
Full brood nest	Yes	Larva/pupa	Yes	Guarding nest
Full-mature brood nest	Yes	Larva/pupa	No	Feeding mature offspring
Mature brood nest	Yes	Adult	No	Feeding mature offspring

Last column is based on^31–33,54^.

### Type of nest founding

We found two types of newly founded nests. Newly excavated nests, which were built by excavating pith from a twig. Discarded nests are the other type. These nests were built from previous nest of *Ceratina* (probably other *C. nigrolabiata* in most cases) by discarding a part of or all original offspring (Figs. S1 and S2). We observed nests of *C. nigrolabiata*, where nest partitions were destroyed and pollen from brood cells was placed on side of the nest. We suppose that original offspring were discarded out of the nest (and on several occasions, we observed discarding of offspring out of the nest). Pollen provisions of the previous nest owner were usually moved to the sides of the nest (Fig. S1). From newly founded nests, 82.69% (86/104) were newly excavated and 17.30% (18/104) were discarded nests. When we counted only nests founded after half of June, the proportion of discarded nests was 22.78% (18/79). From active brood nests, 4.66% (29/622) had apparent relics of usurpation and discarding.

### Presence of parents

#### Newly excavated nests

In newly founded nests, only male was present in 53.48% of nests (46/86, Table 2), only female was present in 10.46% of nests (9/86) and male and female together were present in 36.04% (31/86) nests. Newly founded nests were on average 5.47 cm long (SD = 4.68, range 1–22.1, N = 86). Nests with only male were on average 3.82 cm long (SD = 3.26, range 1.2–16.7, N = 46), nests with only female were on average 5.73 cm long (SD = 4.72, range 1–14.1, N = 9), nests with both male and female were on average 7.85 cm long (SD = 5.49, range 1.9–22.1, N = 31). Nests with both parents were significantly longer than nests with only a male (Tukey HSD test on logarithmic data, difference = 0.6743, *p* = 0.0003), but not significantly longer than nests with only a female (Tukey HSD test on logarithmic data, difference 0.4427 *p* = 0.2256).

**Table 2:** Presence of individuals of parental generation in different nest stages.

Nest stage	No adult	M	MM	F	MF	MMF	MMMF	All nests
Newly excavated nest	0	46	0	9	31	0	0	86
Discarded nest	1	1	0	1	13	2	0	18
Active brood nest	11	35	0	40	527	8	1	622
Full brood nest	494	29	1	102	48	0	0	672
FBN ~ guarded	0	0	0	101	49	0	0	150
FBN ~ orphaned	139	23	1	0	0	0	0	163
FBN ~ plugged	353	6	0	0	0	0	0	359
Full-mature brood nest	102	8	0	48	19	0	0	177
Mature brood nests	68	8	0	8	1	0	0	85

*FBN* full brood nest, *M* male, *F* female.

#### Discarded nests

In 72.22% (13/18) of discarded nests one male and one female were present. Female and two males were present in two nests, only a male was present in one nest, only a female was present in one nest and no adult was found in one nest.

#### Active brood nests

We found male–female pair in 84.72% of nests (527/622), female and two males were found in 1.29% of nests (8/622), female and three males were found in 0.16% (1/622) of nests, no adult was present in 1.76% (11/622) of nests, only male was in 5.6% (35/622) and only female in 6.43% (40/622) of nests.

#### Full brood nests

Most of full brood nests (73.51%, 493/672) were not guarded by any parent (Table 2). When a full brood nest was guarded, then usually by a female (15.18%, 102/672). Only male was present in 4.31% (29/672) and male and female were present in 7.14% (48/672). Males were significantly more often present in nests, where female was also present, than in nests without a female (Chi-square test, Chi = 81.06, df = 1, *p* < 2.2e−16).

#### Full-mature brood nests

Most of full-mature brood nests were not guarded by any adult of parental generation. No parent was present in 57.67% of nests (102/177). If guarding adult was present, it was usually a female in 27.11% (48/177). A pair of male and female was present in 10.73% (19/177) and only male was present in 4.51% (8/177). Interestingly, an old female was present more often in full-mature brood nests than in full brood nests (Chi-square test, Chi = 16.96, df = 1, *p* = 3.826e−05).

#### Mature brood nests

Usually, a nest was not guarded by any adult of parental generation (80%, 68/85). Nests guarded only by male and only by female were present in the same proportion 9.51% (8/85). In one case a nest guarded by male and female together was present.

### Duration of actual male presence

The male found at the time of nest dissection remained in the active brood nest for 10.77 days on average (N = 302, SD = 7.52, range 1–38). In full brood nests, the male found at the time of nest dissection remained there for 4.93 days on average (N = 30, SD = 6.73, range 1–25). However, the difference between nests with male and female couple and nests where a male was present alone was significant (quasipoisson GLM with year and duration of nest observation as covariable, df = 1 and 23 *p* = 4.609e−06, deviance = 67.636, residual deviance = 77.051). Males remained for 7.55 days on average (SD = 7.68, N = 18, range 1–25) in nests, where female was also present, but only 1 day on average (N = 12, SD = 0) in nests where only male was present. In full-mature brood nests, male stayed 3.33 days on average (N = 6, SD = 2.5, range 1–8) at the time of nest dissection. Sample size is too small for testing the difference between nests where a female was present and absent, but we observed both males which stayed in a nest one day and males which stayed multiple days.

### Paternity of nests with small number of offspring

Guarding male was usually not the father of offspring in young provisioned nests. Guarding male was the father of 6.25% (10/160) of all offspring and 9.9% (10/101) of female offspring in nests with 1–3 offspring (N = 70 nests). No offspring was fathered by guarding male in nests with only one offspring (N = 17). The proportion of offspring guarded by it´s own father was 6.2% (2/32) in nests with two offspring and 7.2% (8/111) in nests with three offspring.

### Structure of full brood nest

**Figure 3:**
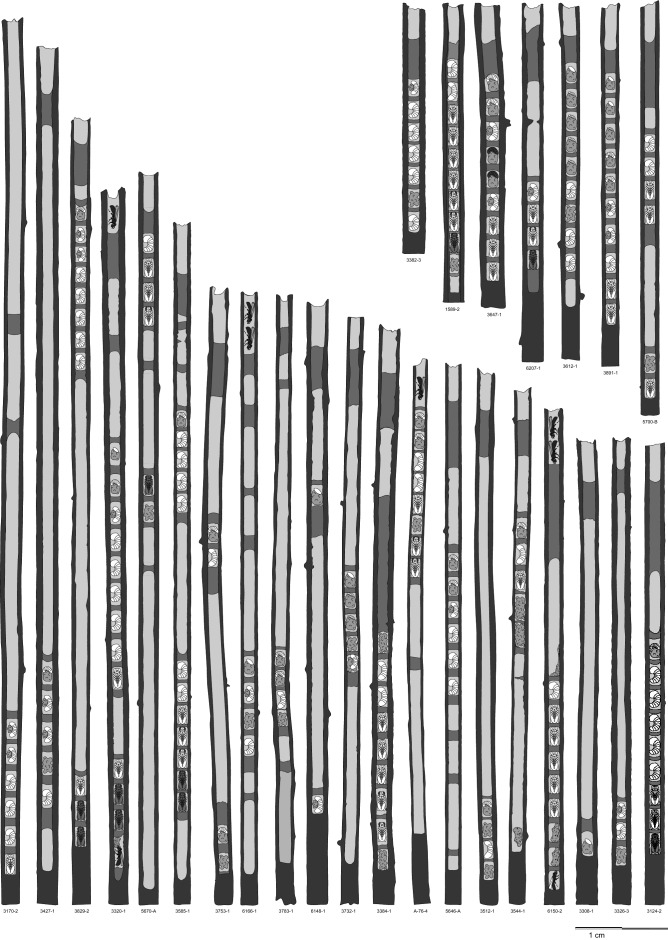
Examples of nests structure of full brood nests of *C. nigrolabiata.* Pictures of all nests are based on real dissected nests.

Full brood nests contained on average 7.59 brood cells (range 1–21, SD = 3.76, N = 566). Empty cells were relatively scarce, but usually present. There were 1.33 (range 0–8, SD = 1.33, N = 530) empty cells per nest on average. Therefore, brood cells were usually adjacent. However, when an empty cell was present, it was usually much longer than a brood cell (Fig. 3). Length of nest was 15.12 cm (N = 670, SD = 4.08) on average and entrance burrow was 3.63 cm long (N = 657, SD = 3.63) on average.

We distinguished three types of full brood nests: (1) guarded nests, (2) plugged nests, (3) orphaned nests. In guarded nests, an old female was present. The Last brood cell was always closed by nest partition. Other two nest types, plugged or orphaned nests, were without presence of an old female. Plugged nests had the last brood cell closed by a filling plug. Filling plug was much thicker than the regular nest partition (Fig. 3) Filling plug was 1.41 cm long (N = 307, SD = 0.87, range 0.2–8.0) on average. Moreover, plugged nests had usually modified nest entrance. All pith between nest entrance and a filling plug was excavated. In orphaned nests, the last brood cell was closed by a regular partition, which was not thicker than regular brood cell partition. Sometimes, the last brood cell was partially provisioned by pollen and was not closed by brood cell partition. This type of brood cell did not contain living offspring.

### Comparison between different full brood nest strategies

The most common type of full brood nest was plugged full brood nest (53.42%, 359/672). The proportion of orphaned (24.25%, 163/672) and guarded (22.32%, 150/672) nests was similar. The number of brood cells was 9.85 on average in guarded nests (SD = 4.14), 6.68 (SD = 3.53) for orphaned nests and 7.06 (SD = 3.30) for plugged nests (Table 3). Number of brood cells was significantly affected by year (Anova, df = 5 and 558, F = 24.418, *p* < 2.2e−16) and also different between nest types (Anova, df = 2 and 558, F = 27.265, *p* = 5.039e−12). However, post hoc tests show that guarded nests are different from plugged (Tukey HSD test, *p* = 0.0000) and orphaned nests (Tukey HSD test, *p* = 0.0000), but there is no difference between plugged and orphaned nest (Tukey HSD test, *p* = 0.1460). Length of nest significantly differed between years (Anova, df = 5 and 659, F = 3.8134, *p* = 0.002059), but did not significantly differ between nest types (Anova, df = 2 and 659, F = 0.4224, *p* = 0.655643). Length of nest entrance significantly differed between years (Anova, df = 5 and 638, F = 7.1525 , *p* = 1.592e−06), and also in full brood nests (Anova, df = 2 and 638, F = 296.7088 *p* < 2.2e−16). Tukey HSD tests showed significant difference between all three nest types. Longest was nest entrance in orphaned nests (mean 7.93 cm, N = 157, SD = 4.67), after it in guarded nests (mean 4.00 cm, N = 148, SD = 3.76) and then the shortest in plugged nests (mean 1.56 cm, N = 352, SD = 1.21, Table 3).

**Table 3:** Comparison between guarded, orphaned, and plugged full brood nests.

Nest type	Guarded	Orphaned	Plugged	All nests
Number of nests analyzed	150	163	359	672
Proportion of nests	0.22	0.24	0.54	1
**Brood cells in nests**				
Mean	9.85	6.70	7.07	7.6
Range	1–21	1–16	1–18	1–21
Standard deviation	4.14	3.53	3.31	3.76
**Empty cells**				
Mean	1.16	0.46	1.84	1.34
Range	0–6	0–5	0–8	0–8
Standard deviation	1.19	0.88	1.33	1.34
**Number of live offspring**				
Mean	7.52	4.98	5.31	5.72
Range	0–21	0–16	0–17	0–21
Standard deviation	4.19	3.32	3.32	3.66
**Length of nest**				
Mean	14.79	15.1	15.28	15.12
Range	4.4–23.4	4.2–26.9	4.3–30.4	4.2–30.4
Standard deviation	3.54	3.8	4.41	4.09
**Length of nest entrance**				
Mean	4	7.94	1.56	3.64
Range	0.5–17.5	0.17–21.3	0.4–15	0.17–21.3
Standard deviation	3.68	4.67	1.22	3.97

**Figure 4:**
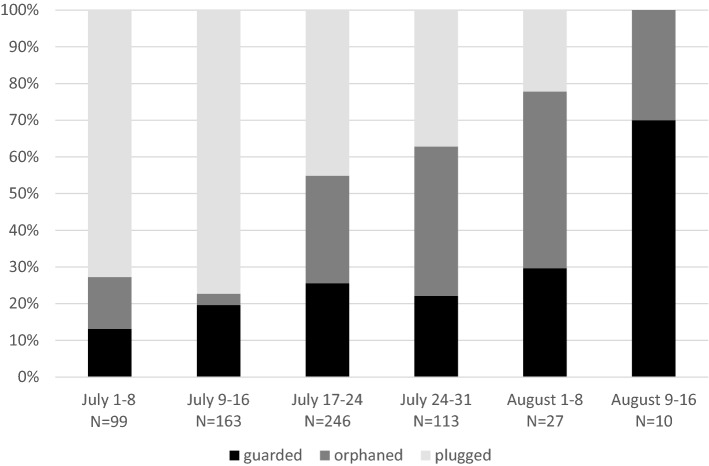
Proportion of types of full brood nests through the season.

There was a distinct difference in the proportion of full brood nest strategies through the season. The proportion of plugged nests was highest at the beginning of full brood nest season (beginning of July) and later decreased. On the other hand, the proportion of guarded and orphaned full brood nests increased from a beginning of July to August (Fig. 4). The proportion of full brood nest types significantly differed between different periods in season (Chi-square test, Chi = 116.87, df = 10, *p* < 2.2e−16).

### Parasites

The most common nest parasites were Ichneumonidae and *Gasteruption*. Both destroyed multiple brood cells and commonly destroyed a large part of a nest. We found an ichneumonid parasite in 6.81% (125/1836) of nests and *Gasteruption* in 2.83% (52/1836) of nests. In 38 cases, we were unable to determine if the parasite is an ichneumonid or *Gasteruption*. When we suppose the same proportion of both parasites in determined and undetermined larvae, we can assume that 8.26% of nests were parasitized by Ichneumonidae and 3.44% by *Gasteruption*. Usually, there was only one larva of these parasites per one nest. We observed 8 cases of two Ichneumonidae larvae in one nest, 2 cases of two *Gasteruption* larvae in one nest and 2 nests where *Gasteruption* and ichneumonid larvae were present together.

Proportions of attacked nests differed between nesting phases. No new founding nests were attacked, as there is no food for the parasite. In active brood nests, only 2.74% were parasitized by Ichneumonidae and only 1.43% by *Gasteruption*. In full brood nests, 11.68% were parasitized by Ichneumonidae and 4.83% by *Gasteruption*. The complete number of parasitized nests is summarized in Table 4.

**Table 4:** Numbers of nests attacked by different groups of parasites.

Parasite	Ichneumonidae		*Gasteruption*		Ichneumonidae and *Gasteruption*	Not identified	Chalcidoid wasps		Conopidae	Malachidae	Unattacked nests	Total nests
Number per nest	1	2	1	2	1 + 1	1	1	2	1	1	NA	NA
Newly founded nest	0	0	0	0	0	0	0	0	0	0	86	86
Discarded nest	0	0	0	0	0	0	0	0	0	0	18	18
Active brood nest	10	0	6	0	0	6	2	0	0	0	598	622
Full brood nest	58	5	24	2	0	22	4	2	0	2	553	672
Full-mature brood nest	15	1	1	0	1	5	0	0	0	0	154	177
Mature brood nest	7	1	3	0	1	0	0	0	0	0	73	85
Other burrows	25	1	14	0	0	5	0	0	1	1	129	176
Total number	115	8	48	2	2	38	6	2	1	3	1611	1836

Ichneumonidae or *Gasteruption* young larvae were not always undistinguished. These parasites are in column not identified.

The proportion of nests attacked by ichneumonid or *Gasteruption* parasites was 16.66% (25/125) for guarded full brood nests, 11.04% (18/163) for orphaned full brood nests and 17.27% (62/359) for full brood plugged nests. Presence of parasite which consumed multiple brood cells (Ichneumonidae or *Gasteruption*) did not significantly differ between years (Binomial GLM, df = 5 and 666, deviance = 35.95, residual deviance = 566.37, *p* = 9.717e−07), and also did not differ between full brood nest types (Binomial GLM, df = 2 and 664, deviance = 3.651, residual deviance = 562.72, *p* = 0.1612). When we excluded nests attacked by an ichneumonid or *Gasteruption* parasite, the proportion of live brood cells differed significantly between years (Binomial GLM, df = 5 and 559, deviance = 33.034, residual deviance = 1291.1, *p* = 3.706e−06) and also between full brood nest types (binomial GLM, df = 2 and 557, deviance = 13.252, residual deviance = 1277.8, *p* = 0.0013255) The proportion of live offspring was 83.41% (N = 127 nests) for guarded nests, 77.92% (N = 141 nests) for orphaned nests and 82.74% (N = 298 nests) for plugged nests.

Other parasites were very rare. We found chalcidoid wasps in 0.44% (8/1836), Malachidae beetles in 0.16% (3/1836) and conopid flies in 0.05% (1/1836) of nests.

### Developmental stage diversity in active brood nests

*Ceratina nigrolabiata* have very fast development in comparison to the rate of provisioning. In 5.14% (32/622) of active brood nests were at least one offspring in the adult stage. Active brood nests, where an adult was present in the innermost brood cell, had a large number of brood cell provisioned (mean 13.71, SD 2.59, range 6–19, N = 29) in comparison to nests, where younger stages were present at the bottom of the nest. Nests with pupa at the bottom had 10.8 brood cells on average (SD-2.84, range 5–19, N = 144). Nests with larva at the bottom had 5.69 brood cells on average (SD = 2.25, range 1–15, N = 268). Nest with egg at the bottom had 2.2 brood cells on average (SD = 1.15, range 1–6, N = 83).

## Discussion

### Role of males

The male–female pair is established in *C. nigrolabiata* before provisioning of brood cells starts. During the period of brood cell provisioning, a pair is present in almost all nests. However, the pair is not stable, and the male may sometimes vanish and is later replaced by another male^32^. However, full brood nests (nests where provisioning was finished) are usually unguarded by any adults, and females are present more often than males. A similar situation occurs in later nest stages. Therefore, males have no important role in brood care after brood cell provisioning is finished and a female stays with her offspring in only a minority of nests.

Biparental care is an uncommon type of parental care in insects^12,15^ and from all bees it is confirmed only in *C. nigrolabiata*^32^. There is an extensive division of labor between males and females in *C. nigrolabiata*. Female does all nest provisioning, but male participates in nest guarding^32^. From other Hymenoptera, biparentality is well documented in several species of the genus *Trypoxylon*^30,31,38^. Males in Hymenoptera are usually short-lived and die shortly after mating^20,39^, therefore, there is a little possibility for performing any care. However, some male participation in care is known also in other Hymenoptera species^26,40^.

Males of *C. nigrolabiata* were present in nests before provisioning started. They were present in newly excavated and discarded nests. New founded nests with male–female pair were more common than nests with only female. Male can help female with nest excavation by throwing filling from a nest or by discarding offspring of previous nest owner. Therefore, males have a partial role with nest building, similarly with males in crabronid wasps from genus *Trypoxylon,* where males help with smoothing of mud using their mandibles^29^.

Although a male is commonly present in the nest before provisioning starts, he is usually not the father of offspring which female laid immediately after she starts provisioning. We found out that the guard male was the father of only 6.25% offspring in nests with 1–3 provisioned brood cells. This proportion is even smaller than the average proportion of offspring guarded by own father which was 10%^32^. Therefore, it is evident that the female mates before provisioning season and the male comes to nest primarily as a stepfather. This situation is in contradiction to other biparental insects, where biparentality is based on monogamy and therefore high relatedness between father and offspring^12^. A minority of offspring (45%) is cared for by father also in the passalid beetle *Odontotaenius disjunctus*^41^*.*

Our results show that males are present in nests where receptive females are also present. They are often in newly founded nest and in almost all active brood nests. However, they scarcely occurred in full brood nests or mature brood nests. In full brood nests, a male was often in nests where a female was also present. Moreover, when the female is removed from active brood nests, the guarding male usually disappears after few days^32^. Therefore, the main male motivation is mate-guarding behavior, not direct offspring care. Males in full brood nests and mature brood nests stay only one or a few days. Therefore, they cannot be fathers of any offspring in the nest. We suppose that two motivations for male presence are possible: (a) mating with newly emerging young females and (b) staying overnight in the burrow in case of single males.

Although male primary motivation is mate-guarding, our previous study shows that male is beneficial for nest productivity^32^. We suppose that presence of a male in the nest is useful as protection of nest when a female is on a foraging trip. In active brood nests is male present in vast majority of nests and when a male is not present, female foraging activity strongly decreases^32^.

Generally, a behavior of males and females of *C. nigrolabiata* is similar to biparental species of genus *Trypoxylon*. In *Trypoxylon*, females also perform all nest provisioning and males stay at the nest entrance and protect the nest against natural enemies^29,38^. Biparental care is supposed to be a by-product of mate-guarding in both groups^31,32^. Some differences between *Trypoxylon* and *Ceratina* exist. In *Trypoxylon*, males stay in the nest entrance head out^38,42^, which allows them to guard more actively than *C. nigrolabiata* males, which block nest entrance by metasoma. Moreover, *Trypoxylon* males usually spent the night outside the nest^29,38^, but males of *C. nigrolabiata* do not leave the nest at night. Generally, we can consider *C. nigrolabiata* and *Trypoxylon* as taxa which convergently developed very similar biparental behavior. Moreover, it is possible that similar behavior occurs also in colletid bee *Leiproctus muelleri*. In this species, males perform nest guarding when female provisions nest^43^. However, more detailed research is necessary for evaluation of the male role in this species. Behavior of males is different in all other hymenopteran groups, in which they assist with caring for offspring. In small eusocial colonies of *Microstigmus nigrophalmus*, males help with nest protection, however there are more males in the nest and they don’t sit in the entrance, but patrol across the whole nest^26^. Male participation on care was detected in some polistine wasps or bumblebees and stingless bees, but the role of males is only small and males help with thermoregulation or food processing^24,25,27,44^. Macrocephalic males were documented in *Lasioglossum* (*Chilalictus*) *erytrurum*, which can guard nest against ants^45^. However, these males were observed in the late phase of nesting, when no brood was produced. Therefore, they probably guard their siblings, not offspring. We suppose that biparental care in Hymenoptera can emerge more easily in species which build linear nests with easy defensible nest entrance, where a male can perform nest guarding. One guarding male is less effective in other types of nest architecture. Male participation on care which emerged in other hymenopteran groups (e.g. eusocial *Polistes* wasps or *Bombus*, macrocephalic males in bees) is not based on pair living. Probably different selection pressures favor its emergence than pressures for typical biparental care which occurs in *Ceratina nigrolabiata* and *Trypoxylon*.

### Alternative nesting strategies

Parental care is costly and reduces future reproduction^1,10,46^. Therefore, animals optimize time when they leave their offspring^46,47^. Most non-eusocial nest-making Hymenoptera abandon the nest after provisioning is finished^20,48,49^, although guarding of nest can substantially increase offspring survival^35,45^. However, guarding of the nest by the female until offspring adulthood is typical for *Ceratina* bees^34,35^.

Our results show that *C. nigrolabiata* has alternative nesting strategies. Some females are trying to guard the nest until the adulthood of offspring. However, most females plug nests by a filling plug and abandon it. This facultative behavior was already documented in *C. chalybea* and *C. chalcites*^35,37^. We suppose that females, which abandoned their nest, build a second nest elsewhere. We do not have direct evidence for this statement, but we found newly founded nests and active brood nests also in late phases of the nesting season (Fig. 2). Moreover, almost all full brood nests were plugged in early phases of nesting season, but guarded nests prevailed in late phases of nesting season. Therefore, we suppose that females usually abandon their early nest(s) and guard their last nest. Females can probably abandon their nest, when there is enough time for second nesting. It corresponds with the semelparity hypothesis. It means that opportunities for reproduction can reduce the extent of parental care^50,51^.

Abandonment of larger brood by mother is generally less probable than abandonment of smaller brood^2,52^. We found out that guarded nests have significantly higher number of brood cells provisioned than abandoned nests. However, we have not detected direct effect of guarding on offspring survival. There was no difference in the proportion of nests attacked by an ichneumonid or *Gasteruption* parasite between guarded and abandoned nests. The proportion of dead brood cells differed between nest types, but was lower only for orphaned and not abandoned nests in comparison with guarded nests. However, the most important cause of brood destruction in case of female removal in *Ceratina* bees is usurpation by other *Ceratina* or the nest destruction by ants^32,35^. This type of attack leads to the destruction of whole or a significant part of nests, but we were unable to detect such effect by a simple comparison of different nest types. Therefore, long-term observations of nest mortality are necessary for comparison of the success of guarding and abandoning strategies.

Guarded and plugged nests differ in the number of brood cells provisioned and length of nest entrance, though the overall difference in nest architecture was small between nest types. In *C. chalybea* and mostly also in *C. chalcites*, the last brood cell was open in guarded nests^32,35^. However, the last brood cell is closed in both nest types in *C. nigrolabiata*. Moreover, last nest partition can be enlarged to filling plug also in some guarded nests (Fig. 3).

Although most of nests without mother are voluntarily abandoned, we detected high proportion of nests (22%) which seems to be orphaned. This is an important difference from *C. chalybea* and *C. chalcites*, where orphaned full brood nests are extremely rare or completely missing^35,37^.

### Natural enemies

*Ceratina* bees are attacked by a wide spectrum of natural enemies. However, the influence of parasitism is usually low due to effective nest protection and short time of larval development^53^. The most common parasites, which we observed, were ichneuomids and *Gasteruption*. Both parasites have a similar effect on nests. Their predacious larvae are much larger than *Ceratina,* and they destroy several brood cells (Fig. S3). The number of broods destructed by one ichneuomonid or one *Gasteruption* is probably about four, but it is difficult to count them as partitions are damaged.

We suppose that the most relevant stage for assessing parasitation is full brood nests. Earlier nest stages had not sufficient time to be parasited. On the other hand, both parasites and *Ceratina* offspring can emigrate from later nest stages, thus full-mature brood nests and mature brood nests aren’t suitable for assessing parasitation. As about 12% of full brood nests were parasitized by Ichneumonidae and 5% by *Gasteruption*, we think that these parasites cause substantial brood loss in this species. On the other hand, other brood parasites were very rare and they probably do not affect *C. nigrolabiata* population substantially.

Nest usurpation plays an important role in *C. nigrolabiata* strategy. From new founded nests, 18.2% were established by usurpation. Moreover, removing experiments show that usurpation by other *Ceratina* bee is the most important reason of failure of the nest with removed female^32^, and most of these usurpers are conspecific individuals of *C. nigrolabiata*. Therefore, interspecific competition plays apparently important role in *C. nigrolabiata*. However, it is a question, why some females frequently abandon nests. Frequency of unguarded nests is even larger than in related species *C. chalybea* and *C. chalcites*^35,37^. In plugged nests is the nest entrance usually excavated, and therefore its usurpation by other *Ceratina* is prevented. It is impossible to guard the nest effectively and therefore plugged nests are probably unattractive for nest usurpation.

### Rate of development

*Ceratina nigrolabiata* have excessively fast development in comparison to the duration of the provisioning period of the nest. Therefore, the largest active brood nests contain already adult offspring at the bottom. Active brood nests with adults at the bottom contained on average more offspring than full brood nests. Moreover, nest with the largest number of brood cells provisioned (23) were not full brood nest but active brood nest with adults at the bottom.

High rate of offspring development leads to less risk of nest abandonment by mother. Adult offspring crawl through nest partitions to the top. They can protect immature siblings against potential intruders soon after mother emigration.

High rate of offspring development complicates determining the average number of offspring in complete nests. The reason is that larger nests are in the stage of full brood nest for a shorter time, which is the only stage when counting of total number brood cells provisioned is possible. When offspring crawl though uppermost brood cell partition, they can emigrate from natal nest. Therefore, average number of brood cells provisioned can be underestimated due to lower probability of detection of a large nest. Moreover, the proportion of guarded nests can be also underestimated, because these nests are larger (and therefore less detectable) on average than plugged or abandoned nests.

## Methods

### General procedures

#### Location

We performed research in Podyjí National Park and surrounding areas (South Moravian Region, Czech Republic), mostly in Havraníky heathland (around coordinates 48°48′32.867"N 15°59′34.963"E). We performed research in 2013–2018.

#### General design

We dissected nests from artificial nesting opportunities. Some of these nests were used also for other experiments (partially published in^32^), but here we present different aspects of *C. nigrolabiata* biology than in our previous paper.

#### Preparation of nesting opportunities

We studied *C. nigrolabiata* nests from artificial nesting opportunities. We used twigs of *Solidago canadesnis*, *Echinops spareocephalus*, *Helianthus tuberosus,* and *Tanacetum vulgare*. We cut twigs to 30–50 cm long fragments. Twenty of these fragments were tied together into one sheaf. Each sheaf was fixed by a bamboo rod to ground. The sheaves were installed before nesting season (April or early May). We established about 1000 sheaves, which corresponds to 20,000 nesting opportunities each year. Therefore, we established around 120,000 nesting opportunities during the whole research.

#### Nest dissection

Nests were collected throughout nesting season from May to September. In total we collected 19 nests in May, 149 in June, 1413 in July, 244 in August, and 11 in September. We dissected most nests in July, since the most important nest stages occur during this part of nesting season. Nests were collected in the evening (after 18 h CEST) to ensure that all inhabitants were present inside the nest. Nests were stored in a refrigerator between the time of collection and dissection. Each nest was open by a knife or clippers. Following parameters were noted for each nest: length of the nest, length of nest entrance, number and stage of immature individuals, number and sex of adult individuals, presence of parasites. Position in a nest was noted for each individual. We also noted the presence of nest partitions, which separated brood cells. We specifically noted presence of a filling plug (enlarged the last brood cell partition, which is usually about 1 cm long).

#### Nest stage classification

We classified nests into categories. Earliest occurred new founding nets (Fig. 1, Table 1), which contained burrows with only adult individual(s) and no pollen ball or provisioned brood cells. We divided such nests to two sub-categories: newly excavated nests, which were newly established, and discarded nests, which were established in twigs that already housed another *Ceratina* nest. In other words, the nest was usurped. Active brood nests contained a pollen ball in the outermost currently provisioned brood cell or an egg in the outermost closed brood cell. Moreover, these nests were not closed by a filling plug. Therefore, active brood nest is a nest, where provisioning of new brood cells is present at the time of nest dissection. Full brood nests contained a larva or pupa in the outermost brood cell, and the partition of the outermost brood cell was still undisturbed. If young adults were present in the nest, they did not crawl through the outermost partition. Full brood nests are nests, where provisioning is already finished, but young adults still did not disturb nest partitions. Therefore, nests at this stage are the most relevant for assessing nest structure. Only these nests are relevant for counting brood cells, because active brood nests are incomplete and young adults can emigrate from full-mature or mature brood nests. Full-mature brood nests had disturbed the outermost brood cell partition (mature adults probably crawled out through this partition) and contained at least one immature offspring. Mature brood nests contained only mature offspring and no juveniles.

We analyzed 1,836 nests of *C. nigrolabiata*: 86 nests were newly excavated, 622 were active brood nests (460 of them were already used for same analyses published in^32^), 672 were full brood nests, 177 were full-mature brood nests, 85 were mature brood nests, 18 were discarded nests, and 176 nests were impossible to classify as any standard category.

Nests, which were impossible to classify into a standard category, were old burrows used to stay overnight or for hibernation (N = 73). These burrows were excavated by a young adult at the end of season or commonly contained evidence of activity of other arthropods (brood cells of other Hymenoptera, spider net) at the bottom. Other nests were evidently build by *C. nigrolabiata* (N = 83), but have not contained any live adult or juvenile *C. nigrolabiata* individual. They contained excrements, dead offspring or only parasites. Another nests (N = 14) contained living *C. nigrolabiata* offspring, but they were distinctly damaged by natural enemies and therefore it was impossible to determine their stage. Last non-standard type of nests were nests which contained only brood cell partitions built by *C. nigrolabiata*, but without living or dead offspring (N = 6). These nests resembled full brood nests, but contained only empty cells (no provisioned brood cell was present).

### Analyses

#### Phenology

We calculated the proportion of types of nests through different parts of the nesting season. Nests dissected in September (N = 11) were excluded from analysis, due to the small number of nests dissected in this period. Therefore, we included 1825 nests. We calculated the proportion of each nest type in each month.

#### Type of nest founding

We distinguished two types of newly founded nests (nests where provisioning did not begin): newly excavated nests which were built as a new burrow, and discarded nests which originated from a previous *Ceratina* nest with its original content discarded by new owners (Fig. S1). We calculated the proportion of both types of nest founding. Moreover, we checked active brood nests and noted evidence of previous discarding (pollen attached on the sides of the nest, Fig. S1).

#### Presence of parents

We calculated proportion of nests where male, female, male–female pair or no adult of parental generation was present. We also counted the proportion of nests where more than one adult of one sex of parental generation was present (In full-mature and mature brood nests were also present young adults. Old and young adults can be distinguished by wing wear). We tested differences in the proportion of nests guarded by different sex by chi-square test. In new founded nests, we tested difference in nest length between nests guarded by a male, a female, or a pair. Firstly, we tested difference by Anova and later we used Tukey HSD tests for pair comparisons. All statistical analyses were performed in R software^54^.

#### Duration of guarding of current male

We measured how long was a nest guarded by a male which was present at the time of nest dissection. We performed this analysis in years 2013–2016. For this experiment, we used all nests in one sector of studied locality. We daily checked the presence and identity of guarding male. We observed nests during the day (between 9 and 17 CEST), when observation of male is easiest, because he stays near nest entrance. We checked each nest once each day. When we found an unmarked male, we marked him with an oil dye. When we found a marked male, we noted his color. Therefore, we were able to determine how long the male is present in the nest. We performed marking of nests through whole provisioning season, from about mid-June to the end of July. We randomly selected nests for nest dissection through this period. Nest stage was determined at the time of dissection. We examined 302 active brood nests, 30 full brood nests, and 6 full-mature brood nests. In this analysis, we included only nests which were observed for at least ten days, though most nests were observed for a longer time. Active brood nests were observed on average for 20.9 days (range 10–41). Full brood nests were observed on average for 25.1 days (range 13–37). Full-mature brood nests were observed on average for 28.2 days (range 17–46). In 27% (83/302) of active brood nests and 3.3% (1/30) of full brood nests, the same male was present throughout the whole observation period. Therefore, the duration of actual male presence is underestimated in active brood nests and probably correctly calculated in full brood nests and mature brood nests. For a detailed description of this method, see^32^. For full brood nests, we tested the duration of presence of currently guarding male between nests where a male was alone and where a male was in pair with a female. We used quasi-Poisson GLM with year and duration of observation period (in days) as covariable. Quasi-Poisson model was used because overdispersion was present. Analysis was performed in R software^54^.

#### Paternity in small nests

For this analysis, a subset of nests analyzed in previous study was used^32^. Procedures of DNA isolation, microsatellite genotyping, and paternity analysis are described in^32^. Nests were selected for analysis according to these characteristics: (1) Nests had between one to three provisioned cells with offspring, (2) guarding parent pair was present, (3) all offspring in the nest was offspring of guarding female. 17 nests with one offspring, 16 nests with two offspring, and 37 with three offspring were included in this analysis. In these nests, we tested paternity of the guarding male.

#### Full brood nest structure

We calculated the number of provisioned cells, empty cells, and living offspring for full brood nests and active brood nests. The number of provisioned cells was possible to calculate only in nests which were not influenced by an ichneumonid or *Gasteruption* parasite, or only slightly influenced. These parasites destroyed multiple brood cells, and it was impossible to determine the precise number of brood cells destroyed. The number of empty cells was possible to be calculated in nests which were not parasited by *Gasteruption* or Ichneumonidae, and if any adult offspring destroyed partitions in the bottom part of the nest. When adult offspring destroyed brood cell partition of at least one brood cell, it was impossible to determine if an empty cell were destroyed or not.

#### Comparison of different full brood nest types

Full brood nests were classified in three categories: guarded, plugged, and orphaned (Table 1, Fig. 1). Guarded full brood nests contained an old adult female in the nest entrance. Last brood cell was always closed. Plugged nests did not contain female at nest entrance and were closed by a filling plug (nest partition on average 1.41 cm thick, much more than regular brood cell partition). Last brood cell was always closed. Orphaned nests did not contain female at nest entrance and the last brood cell partition was of the same thickness as the other partitions in that nest. Sometimes, the last brood cell was opened, without living offspring and only partially provisioned by pollen in orphaned nests. Adult males can be present in nest entrance of all types of these nests. We did not use the presence of male as a factor for nest classification. We tested differences in nest features between three nest categories using Anova tests as a covariable. We used equation with interaction (dependent variable ~ year * nest type) or without interaction (dependent variable ~ year + nest type). The decision to include the interaction or not was based on model AIC. Later, we used TukeyHSD pair comparisons when significant Anova results were found. The statistical analyses were performed in R software 3.6.1^54^.

#### Parasitism

We analyzed the presence and number of natural enemies at the time of nest dissection. We were unable to distinguish between early stages of ichneumonid and *Gasteruption* larvae. Therefore, we inserted these larvae in Eppendorf tubes and tried to rear them at least to prepupal stage when these two parasites are easy to distinguish. Some larvae died before this stage (N = 38). For assessment of total parasitation rate, we divided these cases proportionally between ichneumonids and *Gasteruption*.

We tested the association between presence of parasites which attack multiple brood cells (ichneumonids and *Gasteruption*) and full brood nest type (guarded vs. plugged vs. orphaned) by chi-square test. Moreover, we tested differences in proportion of brood cells with dead offspring between full brood nests types. For this analysis, we excluded nests parasitised by an ichneumonid or *Gasteruption* because the number of damaged brood cells was not easy to count. We tested this difference using binomial generalized linear model because the proportions of dead brood cells have binomial distribution. We used year as covariable. We used equation with interaction (dependent variable ~ year * nest type) or without interaction (dependent variable ~ year + nest type). The decision to include the interaction or not was based on model AIC. The statistical analyses were performed in R software 3.6.1^54^.

#### Analysis of developmental stage diversity in active brood nest

For active brood nests, we noted the stage of offspring in the innermost (= oldest) brood cell. We calculated the proportion of nests with adult, pupa, larva, and egg in the innermost brood cell. Moreover, we calculated the average number of brood cells for active brood nests with each offspring stage separately.

## Supplementary Information

Supplementary Information 1.

Supplementary Information 2.

## Data Availability

All relevant data are attached in XLS file as supplementary material.
