# Supplementary material for: Nesting of Ceratina nigrolabiata, a biparental bee
Source: Sci Rep. 2021 Mar 3;11:5026. doi: 10.1038/s41598-021-83940-4 (PMC7930280; doi:10.1038/s41598-021-83940-4)
Supplement: Supplementary file 1 — Supplementary Information 1. [file 41598_2021_83940_MOESM1_ESM.docx]

**Nesting of *Ceratina nigrolabiata*, a biparental bee**

Michael Mikát*, Eva Matoušková, Jakub Straka

Department of Zoology, Faculty of Science, Charles University, Prague

* Corresponding author: Michael.mikat@gmail.com

**Supplementary materials**

**Supplementary Figures:**


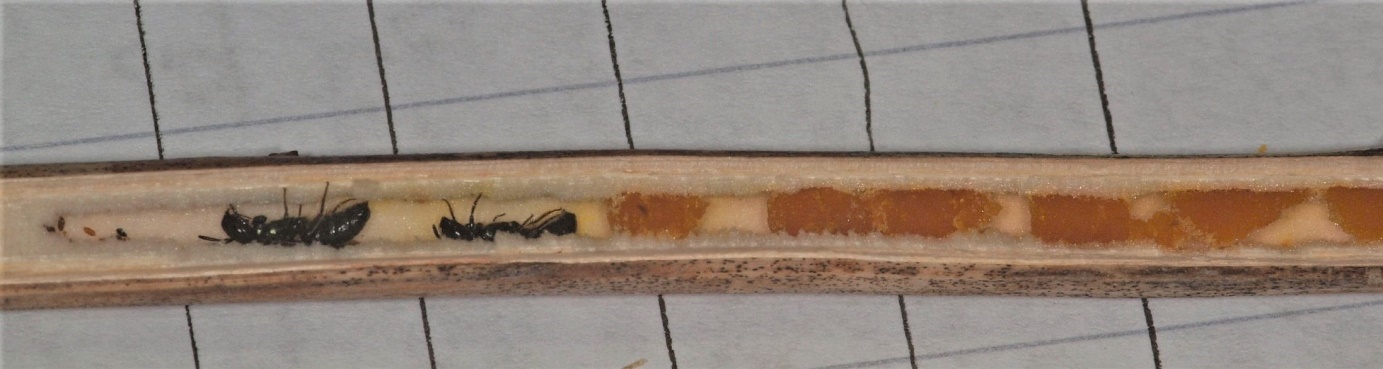


Fig. S1: Nest establishment by discarding. Female and male discard offspring of previous owner. Pollen is stacked on the wall of nest and maybe reused.


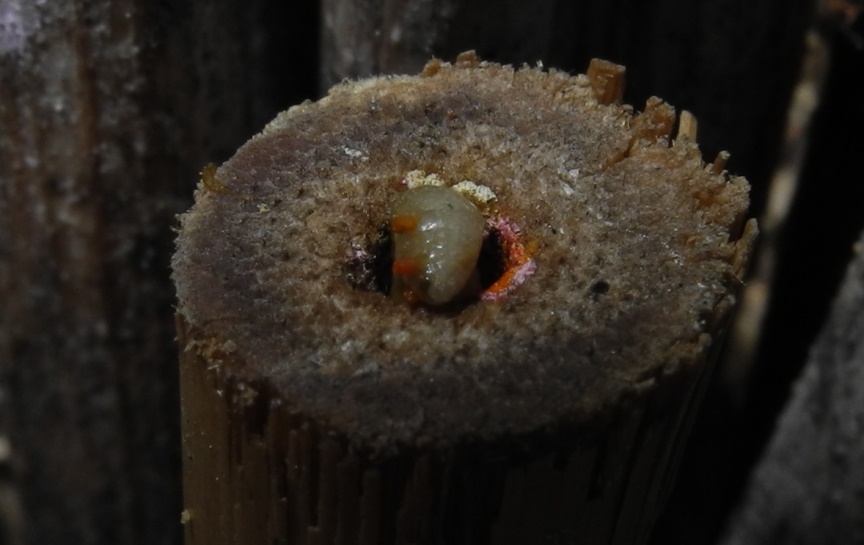


Fig. S2: Discarding of *C. nigrolabiata* larva


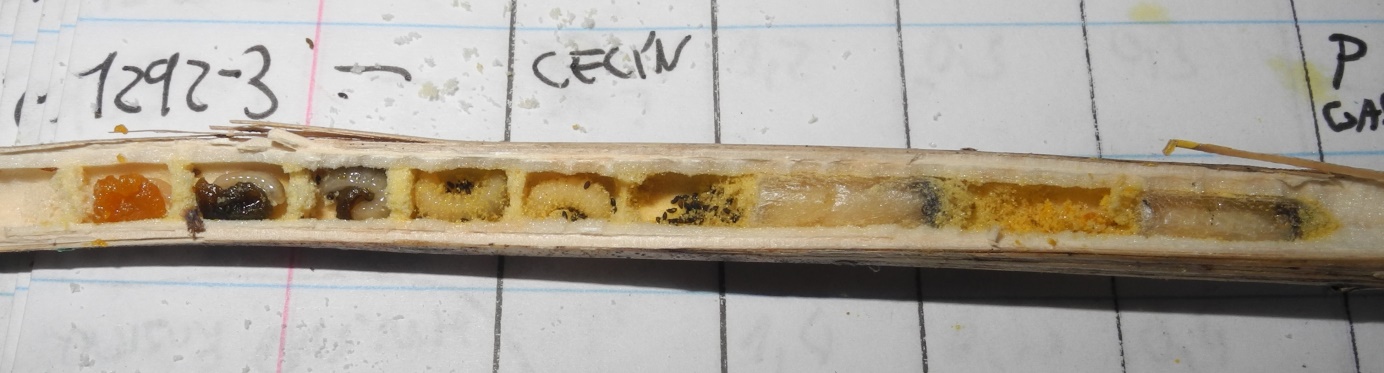


Fig. S3: Nest parasited by two ichneumonids. Each larva destroyed multiple brood cells. Outer brood cells were not attacked. Photo: Lukáš Janošík.
